# Vaccine rollout strategies: The case for vaccinating essential workers early

**DOI:** 10.1371/journal.pgph.0000020

**Published:** 2021-10-13

**Authors:** Nicola Mulberry, Paul Tupper, Erin Kirwin, Christopher McCabe, Caroline Colijn

**Affiliations:** 1 Department of Mathematics, Simon Fraser University, Burnaby, Canada; 2 Institute of Health Economics, Alberta, Canada; 3 Health Organisation, Policy, and Economics, School of Health Sciences, University of Manchester, Manchester, United Kingdom; 4 Faculty of Medicine and Dentistry, Department of Emergency Medicine, University of Alberta, Alberta, Canada; FIOCRUZ: Fundacao Oswaldo Cruz, BRAZIL

## Abstract

In vaccination campaigns against COVID-19, many jurisdictions are using age-based rollout strategies, reflecting the much higher risk of severe outcomes of infection in older groups. In the wake of growing evidence that approved vaccines are effective at preventing not only adverse outcomes, but also infection, we show that such strategies are less effective than strategies that prioritize essential workers. This conclusion holds across numerous outcomes, including cases, hospitalizations, Long COVID (cases with symptoms lasting longer than 28 days), deaths and net monetary benefit. Our analysis holds in regions where the vaccine supply is limited, and rollout is prolonged for several months. In such a setting with a population of 5M, we estimate that vaccinating essential workers sooner prevents over 200,000 infections, over 600 deaths, and produces a net monetary benefit of over $500M.

## Introduction

As of March 2021, several vaccines have received widespread approval for use against COVID19 [1]. Vaccination rollouts are underway in much of the world, but the quantity of vaccine doses available differs greatly between jurisdictions. The question of how to deploy vaccines, taking into account their efficacy in preventing symptomatic disease, their efficacy in blocking transmission, and the demographics and underlying contact structure of the population, poses substantial and ongoing challenges. Many jurisdictions are using primarily age-based rollout strategies, where the oldest are vaccinated first and the youngest last, with the rationale that the risk of severe outcomes from COVID-19 steeply increases with age. Such strategies may appear optimal if we are only considering the ability of the vaccines to prevent illness, and/or if everyone is likely to be exposed.

Data on the reduction of infection and transmission from vaccination are accumulating as more countries deploy vaccines. Recent phase 3 trials have shown that the Moderna vaccine [2], the PfizerBioNTech vaccine [3], and the AstraZeneca [4] vaccine are effective at preventing symptomatic infection and severe illness. There are broadly two ways that a vaccine can attain this kind of result: either by preventing infection from occurring in the first place (known as “sterilizing immunity”) or allowing infection but preventing disease [5]. In the first case the vaccine necessarily prevents onward transmission, but in the latter case the vaccine may or may not prevent subsequent transmission, depending in part on whether it decreases viral load. The emerging data show both a high rate of sterilizing immunity and a reduction of viral load in the minority of those vaccinated who do become infected. A 2/3 reduction in infection was observed in *asymptomatic* infection [6], which together with the already documented reduction in symptomatic cases [2] gives a high overall reduction in infection. Similar preliminary results are emerging for the Pfizer vaccine [7,8] and for the AstraZeneca vaccine [9]. As well, substantial reduction in viral loads have been found among those who become infected after receiving either the AstraZeneca vaccine [10] or the BioNTech/Pfizer BNT162b2 vaccine [11,12], suggesting reduced transmission even when infection does occur.

A natural goal for minimizing the impact of the pandemic is to prevent as many deaths due to COVID-19 as possible. This is a primary motivation for vaccination plans that start with the oldest individuals and then go down through the age cohorts, since risk of mortality increases sharply with age [13]. But another important consideration is that, in a small but significant number of cases at all ages, COVID causes long-lasting symptoms that can be debilitating [14]. There is a syndrome that has come to be known as *Long COVID* [15]: extreme fatigue and other COVID symptoms that may last for weeks or months [16], and may turn out to be chronic to the best of our knowledge now [14]. The symptoms are similar to those described by survivors of SARS [17], and fit the clinical definition of myalgic encephalomyelitis/chronic fatigue syndrome (ME/CFS) [18]. In addition, there are *COVID complications* [19]: severe secondary conditions caused by COVID-19 infection, including diabetes [20], organ damage [21,22], and neurological and psychiatric disorders [23]. These can also be caused by other severe viral infections such as SARS [17,24] and Zika virus [25,26], and early evidence suggests that they are not rare for hospitalized COVID cases. Both of these outcomes, Long COVID and COVID complications, which we will together refer to as *chronic outcomes*, are likely to be lasting and serious consequences of the pandemic for many individuals [27]. Accordingly, vaccination strategies need to factor them into consideration, despite uncertainty in the duration, severity, and frequency of their occurrence.

There is an emerging literature on optimizing vaccine rollout for COVID-19 given limited vaccine resources. An important early study by Bubar et al [28] modeled the pandemic using an age-stratified SEIR model and compared five vaccine prioritization strategies. They found that targeting 20–49-year-olds reduced the overall number of infections, but led to higher mortality among the elderly. Their results stem from the conditions under which their model is run, which allow for a higher prevalence than has been reached in many jurisdictions where non-pharmaceutical interventions (NPIs) are deployed before hospital capacity is exceeded. Matrajt et al [29] use modelling to explore age-based vaccination during vaccine rollout with no NPIs in place, similarly therefore not accounting for reductions in transmission due to either ongoing or repeatedly-introduced measures reducing case numbers. Both these studies [28,29] do not include essential worker categories. Another approach is that of Chen et al [30] who used an agent-based model with a detailed social contact network to study vaccine prioritization, with Virginia as an example. They found that targeting individuals with many contacts rather than a purely age-based strategy lead to substantially better outcomes. Jentsch et al [31] also do not consider essential workers, but supports vaccinating younger age groups earlier under some circumstances. Other studies are also split, and favour vaccinating essential workers early under some circumstances [32,33] or a primarily age-based prioritization in others [34].

We reassess vaccine rollout strategies in the light of the emerging data on vaccine efficacy and in light of the importance of chronic outcomes of COVID-19 infection. We compare age-based vaccine rollouts with strategies that prioritize those we call essential workers. Throughout this work, we use the phrase “essential workers” to mean those who have high contact at work. This is distinct from “essential services” and could include teachers, taxi drivers, retail workers, food production workers, law enforcement and public safety, first responders, social workers, agriculture, transportation and many more [35]. Our analysis is differentiated by our contextualisation to settings where COVID prevalence is maintained by NPIs, our consideration of chronic outcomes of COVID, and our use of the Net Benefit framework.

## Methods

We use an age- and contact-structured compartmental model to investigate the impact of different vaccination strategies in British Columbia, incorporating chronic outcomes of COVID-19 infection, along with infections, hospitalizations and deaths. The model has susceptible, exposed, infectious and recovered individuals and was originally developed to explore vaccination by age, considering “leaky” or “all or nothing” vaccination and taking existing seroprevalence into account [28]. See Section S1 Text in the Supplementary Material for details and parameters of the model. Our model is an extension of the framework introduced by Bubar et al [28], but now considers both age and essential worker status; in addition, we explore Long COVID and chronic outcomes. Furthermore, we attach health economic outputs by applying a Net Benefit framework, including the expected cost due to hospitalization and chronic conditions resulting from infection, as well as decrements in health utility, which are measured using quality-adjusted life years (QALYs).

In total, we model 15 population groups by age and work status {0–9, 10–19, 20–29, …, 70–79, 80+, 20-29^*e*^, 30-39^*e*^, …, 70-79^*e*^}, where the *e* superscript denotes an “essential worker” group. Our model of vaccine efficacy works in two stages: a vaccine may prevent infection altogether, but even when it does not, it may still prevent severe outcomes (symptomatic disease, hospitalization, death and chronic outcomes). We have made the code necessary to reproduce our results publicly available at https://github.com/nmulberry/essential-workers-vaccine.

Our simulation approach is motivated by the vaccination programs in British Columbia, across Canada, and in similar jurisdictions. Such jurisdictions have had a relatively small portion of the population naturally infected at the time of writing, and have begun vaccination with the very elderly during a time when social distancing measures have kept the reproduction number low while those over 80 years of age are vaccinated. We initialize our simulations using reported case counts from British Columbia. In line with observed events, we hold *R* at 1.05 from January 1, 2021 for 60 days while those aged 80+ are vaccinated. After 60 days we raise *R*, typically to 1.15, 1.3, 1.5 in the main text and with some higher examples in the supplement. This rise in transmission models either relaxation of distancing measures, reduced compliance to widespread distancing measures, or rising frequencies of higher-transmission COVID-19 variants of concern [36]. During the next 210 simulated days, we proceed with the specified vaccination scenario. Both among 80+ and the other age groups we model age-specific hesitancy, with some portion of each age group declining the vaccine.

We consider five vaccination scenarios for the period of time after the 80+ age group is vaccinated. In scenario A, available vaccines are distributed to age groups in order of decreasing age. In scenario B, after the 80+ group is vaccinated, the vaccine is distributed to everyone else with no preference for age. In scenarios C, D, and E, after the 80+ group, essential workers are then vaccinated without regard for age. In scenario C, the rest of the population is vaccinated in decreasing order of age. In scenario D, the rest of the population is vaccinated without regard for age. In scenario E, the 70–79 cohort is vaccinated next and then the rest of the population is vaccinated without regard to age. For each scenario, the rate of vaccination is initially about 0.075% population per day until the entire 80+ cohort has been vaccinated. The rate of vaccination then increases to about 0.45% population per day until the end of the simulations (this value is fixed across all scenarios A–D, and sensitivity to this value is explored in S1 Text Supplemental Material (Fig L). These rates were chosen to match the projected timelines released by the British Columbia Centre for Disease Control as of February 2021 [37].

Finally, we considered some economic measures of the cost of the pandemic from a health system payer perspective: health utility losses measured in QALYs lost and net monetary benefit (NMB). Estimating health utility in terms of QALYs allows us to quantify and compare loss of quality and duration of life due to illness, disability, and death [38]. QALYs are estimated such that the maximum value of 1 indicates a year in perfect health, whereas a value of 0 indicates no health (death). Utility decrements due to acute COVID infection, hospitalizations, chronic outcomes, and death are estimated as the difference between expected health, and health utility due to COVID. A key parameter in our estimates is the standardized mortality ratio (SMR): the increased hazard of dying COVID patients have of non-COVID causes (on average) relative to other patient of a similar age.

Following Briggs et al [39], since COVID fatalities often occur to patients with preexisting conditions that may shorten their lifespan, we took an SMR of 2. Details of the calculations are in the S1 Text Supplementary Material. These estimates are synthesized into NMB by converting QALYs to monetary value, to allow a unified way of evaluating vaccination strategies [40].

Following the data from studies on the Pfizer and Moderna vaccines on the impact of vaccination on infection and on viral load, and data from the clinical trials on the impact on severe disease [2,3,6–8], we vary the effectiveness of the vaccine in preventing infection from 60% to 90%, and set the efficacy of it preventing illness and death in the case of infection at 90%. This yields an overall effectiveness at preventing symptomatic infection (including severe outcomes) between 96% and 99%.

## Results

We find that vaccinating essential workers earlier gives large reductions in infections, hospitalizations, deaths, and instances of Long COVID (cases with symptoms lasting longer than 28 days), across a range of scenarios for transmission and vaccine efficacy. Except for deaths, results were similarly good with a strategy that vaccinates younger people sooner without targeting essential workers. However, with scenarios vaccinating the elderly later, they have very slightly higher rates of adverse outcomes, as expected, depending on how they are prioritized.

**Fig 1:**
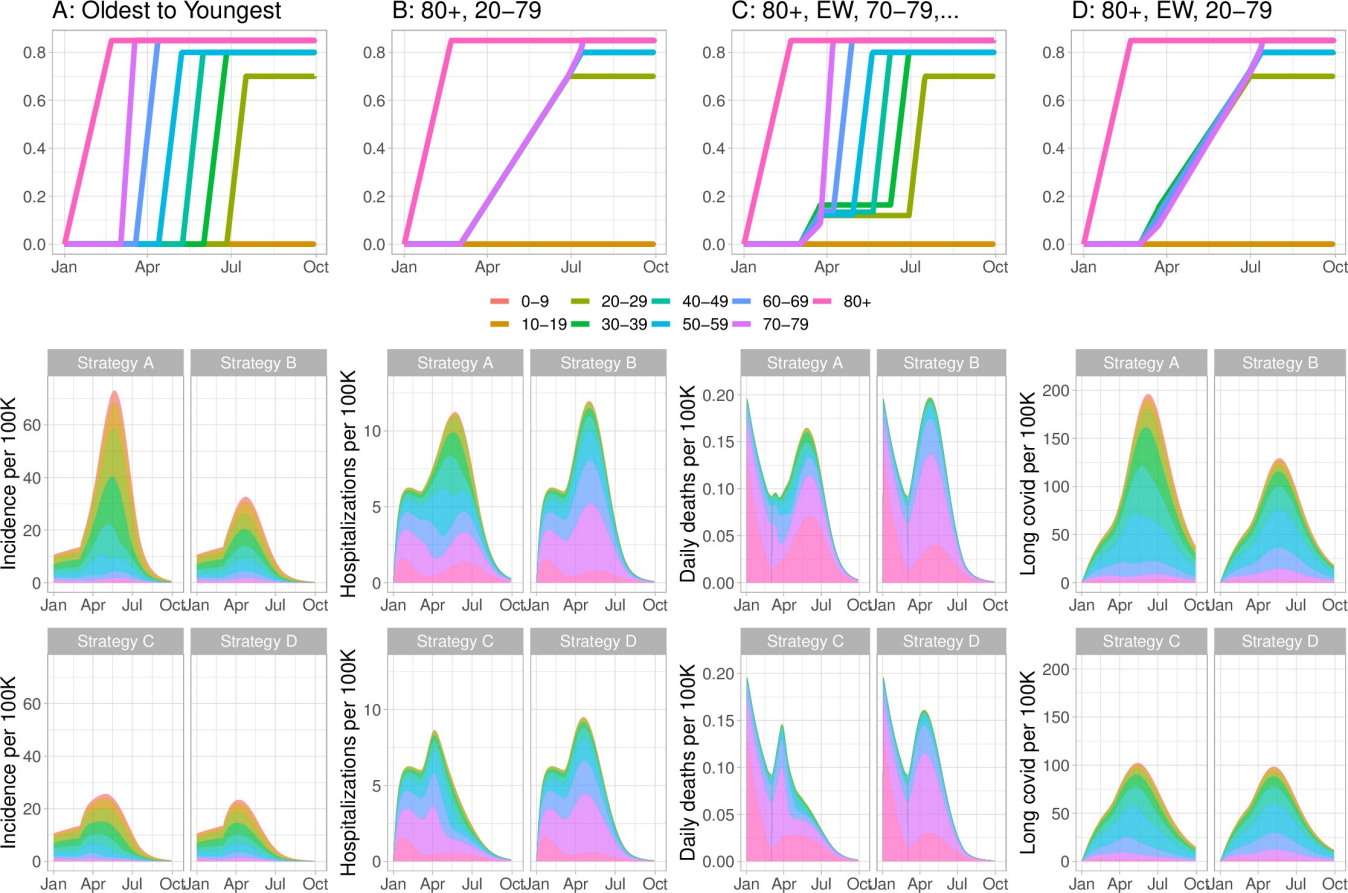
Vaccinating younger adults sooner, and vaccinating essential workers sooner, reduces cases, deaths, hospitalizations and Long COVID (COVID symptoms lasting longer than 28 days). In our model the rollout consists of an initial phase during which those over 80 are vaccinated and *R* = 1.05, followed by a second stage where the transmission rate is higher (*R* = 1.3 here). In these simulations vaccine efficiency is 90% for preventing disease and 75% at preventing infection and therefore subsequent transmission. Top row: Illustration of the vaccination programs by age group over time: Fraction of the age group vaccinated versus time. Next two rows: Simulated cases, hospitalization, deaths and Long COVID prevalence over time.

Fig 1 illustrates the high impact of including younger age groups sooner in the program than in a primarily oldest-first strategy (A), either through expanding to all age groups after those over 80 have been vaccinated (strategy B) or through vaccinating essential workers of any age group after those over 80 and then continuing from oldest to youngest (C), or expanding to all ages after essential workers (D). Vaccinating essential (high-contact) workers early has a strong effect, and can be done within the context of a broader oldest-first vaccination scheme. This does not require additional doses of vaccine. The shading in Fig 1 indicates which age groups are affected in four example simulations in the model, with four distinct vaccination strategies.

**Fig 2:**
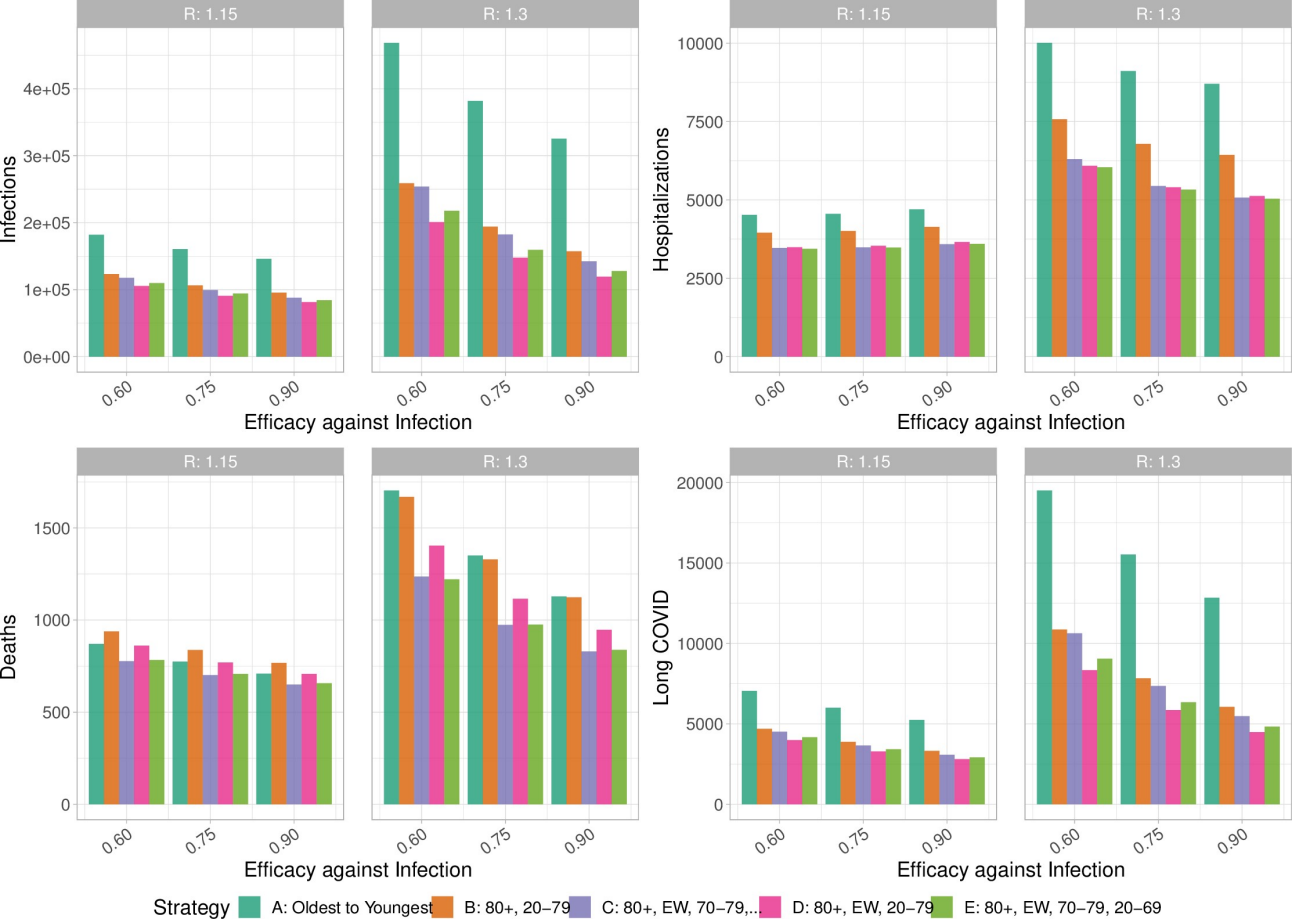
Vaccinating essential workers earlier in the program has benefits for cases, hospitalizations, death and Long COVID (COVID symptoms for longer than 28 days). This does not depend sensitively on the efficacy of the vaccine against transmission, nor on the underlying transmission rate. Similar results are attained by vaccinating over all age groups (strategy B), with the exception that this gives less reduction in deaths.

Fig 2 shows the simulated total cases, deaths, hospitalizations and Long COVID cases for a range of transmission-blocking efficacy and for two values of the underlying *R*. At the time of writing, many jurisdictions are vaccinating on a primarily oldest-to-youngest vaccination program [41–43], although some health care workers and staff in long term care homes and some other vulnerable communities are included in the early stages. In our model, a strictly age-based approach leads to considerably more cases and more Long COVID across a range of values of the vaccine’s efficacy against transmission and across a range of *R* values. When *R* is kept very low (1.15), for example through continued strong social distancing, all of the simulated strategies do well at reducing deaths. When *R* rises to 1.3, strategies placing essential workers after 80+, either continuing with an age-based rollout or opening to all adults aged 20–69 after those 70+, have an advantage in reducing deaths in addition to strong advantages for infections, hospitalizations and Long COVID. Other differences between the age ordering are very small compared to the difference between “oldest first” strategies and *any* alternative that prioritizes essential workers or even all younger adults earlier in the program.

In S1 Text Supplemental Material we explored the sensitivity of our vaccine strategy comparisons to the portion of workplace contacts taking place among essential workers (Fig G), the efficacy against transmission (Fig H), the portion of workers considered “essential” (Fig I) and to the contact matrix (Fig J). We explore a wider set of strategies (Fig B). Consistently, vaccinating essential workers earlier has considerable benefits compared to oldest-first strategies while the relative merits of different age-based rollouts depend on assumptions. For example, when the portion of workplace contacts among nonessential workers is higher, strategy C (80+, EW, 70–79,..) which is predominantly oldest first loses some of its benefit because essential workers have less of the overall contact (Fig G). While the efficacy against transmission has a high impact on the outcomes, it does not much impact the relative performance of the strategies unless *R* is high (Fig H). Finally, we determine the optimal strategy simply from the point of view of minimizing deaths; despite the fact that the mortality risk motivates oldest-first vaccination, this strategy is only the best for deaths as an outcome when efficacy against transmission is extremely low (0.1–0.2) and when *R* is high (Figs K and L). See the S1 Text Supplemental Material for further details.

**Fig 3:**
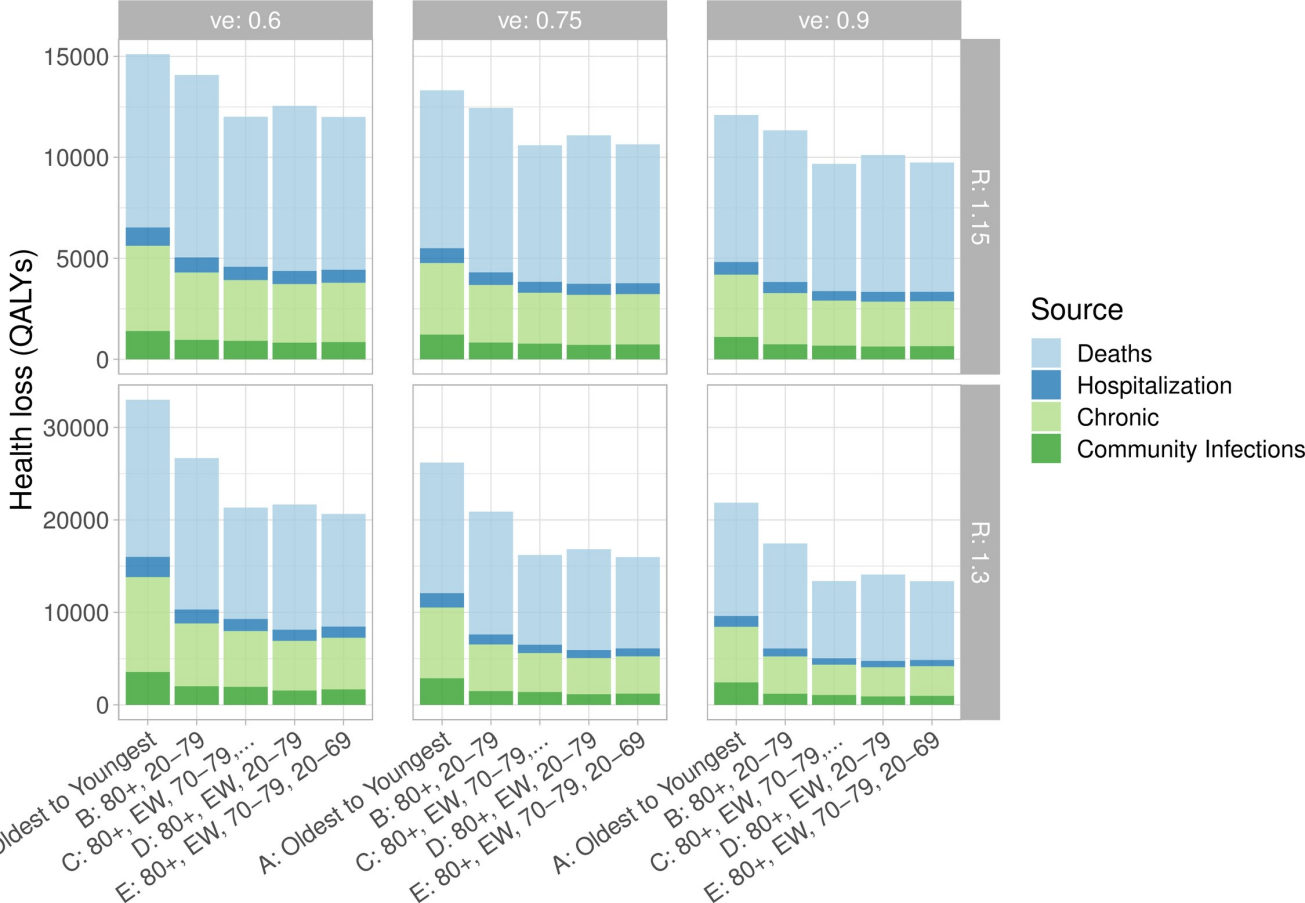
Health losses (QALYs). *R* and the efficacy against transmission vary and are labelled on the side (*R*) and the top (efficacy). Community infections refers to QALYs lost from acute COVID infections that do not result in hospitalization.

Fig 3 shows health utility losses in QALYs and their source (cases, chronic impacts, deaths and hospitalizations). Here chronic impacts includes both chronic Long COVID symptoms (similar to ME/CFS) and other long-term complications. Deaths are naturally a large contribution to health utility loss; the next biggest contribution by far are the chronic impacts where we find that vaccinating essential workers sooner has profound benefits For example, if *R* rises to 1.3 and the vaccine is 75% effective in preventing transmission, the combined reduction in deaths, chronic impacts, cases and hospitalizations when essential workers are vaccinated after those aged 80+ means that over 11000 QALYs are gained (Fig 3, bottom middle panel). Of these, 3000 are due to chronic impacts. When efficacy against transmission is lower, because there are more infections, it remains very beneficial from a health utility point of view to vaccinate essential workers sooner. As with hospitalization, deaths and Long COVID, the differences between age rollouts beyond whether essential workers are included early is relatively small.

**Fig 4:**
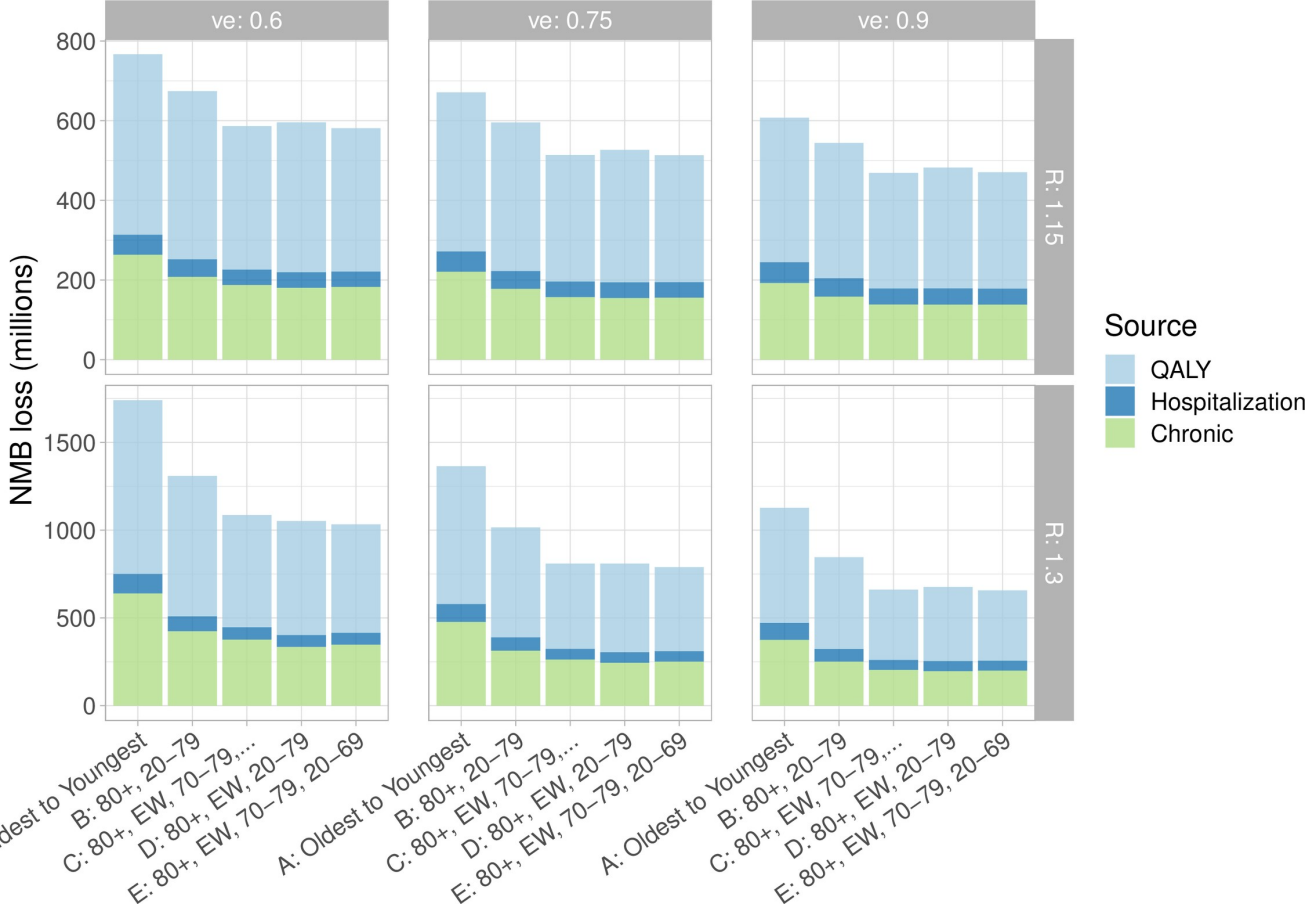
Net Monetary Benefit (loss), due to the cost of treating chronic outcomes, hospitalizations during the pandemic, and health utilty converted from QALYs lost shown in Fig 3. Transmission-blocking efficacy *ve* and *R* vary as labelled over the panels.

We find similar effects when we estimate the incremental costs of the pandemic including both direct costs of hospitalization and chronic outcomes and health utility decrements converted to monetary values (Fig 4). Vaccinating essential workers sooner reduces the overall NMB loss due to the pandemic by 50 to 65%. This results in a potential savings of (for example) over $400M (if *R* = 1.3); see Fig B in the S1 Text Supplemental Material. In all scenarios, NMB lost was largest for the age-based immunization strategy (A). The largest improvement in NMB was achieved with strateges C, D and E, irrespective of vaccine efficacy. Fig 4 highlights the potential for a substantial impact of chronic consequences of infection, both related to health utility losses, and future health system cost.

**Fig 5:**
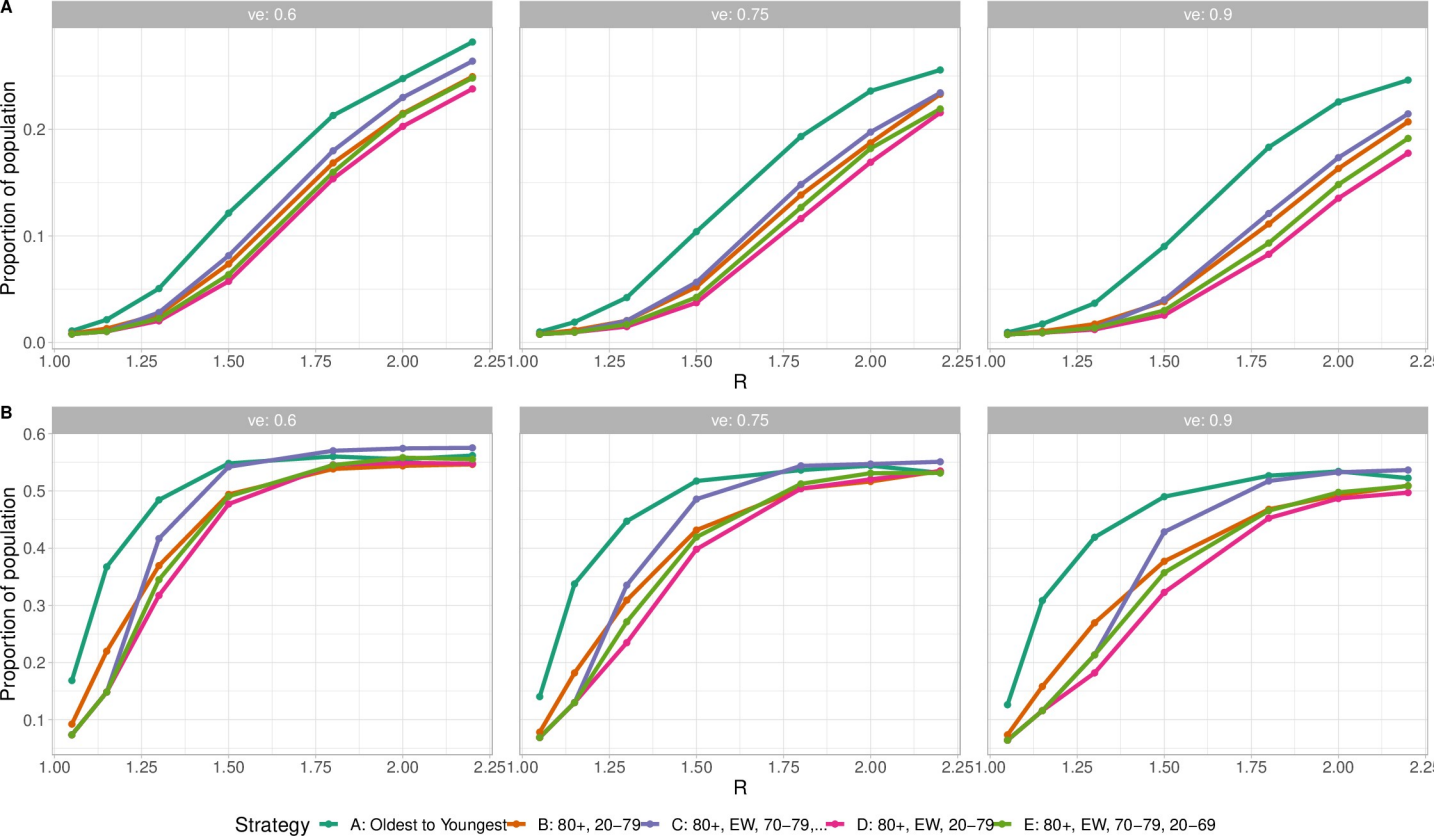
Immunity and vaccination at the time when infections begin to decline (turn around time), as a function of *R*. (A) Proportion ever infected by turn around time. (B) Proportion ever infected or vaccinated by turn around time. The efficacy of vaccination against infection (*v*_*e*_) is varied from 0.6 (left) to 0.9 (right).

Finally, we explore immunity at the point where transmission begins to decline (while keeping *R* constant through, for example, NPIs). Fig 5 shows the fractions of the population who were infected (top panels), or who were protected by either natural infection or vaccination (bottom panels), at the time when simulated infections began to decline, for three transmission-preventing efficacies and for a range of *R*. Consistently, the oldest to youngest strategy requires more immunity than strategies vaccinating essential workers or younger people sooner.

## Discussion

Strategies that vaccinate essential workers early lead to substantial reductions in the number of infections, hospitalizations, deaths, and cases of Long COVID relative to a strategy of oldest first. Essential workers cannot effectively isolate under any social distancing regime. Our finding that there is a strong benefit across many outcomes if these groups are vaccinated early hold for any group of similarly high-contact individuals, and is consistent with Centre for Disease Control recommendations for vaccination of essential workers [44]. Vaccinating essential workers is important from an equity standpoint, as the COVID-19 pandemic has disproportionately impacted essential workers. Across neighbourhoods in Toronto, for example, per-capita COVID cases and deaths were 2.5 to 3 times higher in neighbourhoods with high (vs low) concentrations of essential workers [45]. Essential workers often have lower incomes, and may be hired as contractors; they may not have paid sick leave, and may have limited ability to negotiate safe working conditions [45]. While many others are able to work safely at home, these individuals cannot. Our findings suggest that prioritizing them for vaccination would help to reduce this substantial disparity, and would not come at a cost of increased adverse outcomes.

We have considered contexts in which interventions are brought in to reduce case numbers when the health care system begins to be stretched for capacity, as has occurred in most jurisdictions in Canada, the US, and Europe (i.e. *R* is not left much higher than 1 for long periods). Larger transmission rates lead to the rapid overwhelming of the healthcare system, and governments then introduce lockdown or stricter social distancing measures in response. For example, in the United Kingdom, when cases reached a rolling average of 55,000 new cases per day (approximately 80 per 100K at the end of 2020), strict lockdowns were implemented with 78% of the population under severe restrictions [46]. Across Canada, lockdown measures were introduced when daily incidence reached approximately 20–50 cases per 100K. Sustaining *R* = 2 before vaccination rollout is at a level to substantially protect the population results in incidence 40–60 times that, or at least 10 times the UK value. Even at a reduced effective hospitalization rate (with older groups vaccinated), health care systems would be overwhelmed if these incidence levels were reached. Accordingly, *R* is not likely to remain high enough, in advance of vaccination, for oldest-first strategies to be best; distancing and other measures have consistently been put in place to prevent this.

The mechanisms behind our results are illustrated in Fig 5, which shows that the oldest to youngest strategy requires the most immunity (through either infection or vaccination) out of all strategies considered. This is because individuals who have a comparatively low likelihood of exposure and transmission are vaccinated, but their protection contributes less to the population’s collective protection than vaccination of those who are at risk of exposure and transmission. Accordingly, vaccinating 80+, essential workers and then all ages requires the fewest infections and the least immunity in order for cases to decline; in this strategy those likely to transmit are protected efficiently. The immunity required for infections to decline is more sensitive to the vaccination strategy when *R* is relatively low; above *R* = 2, the required protection for all strategies is just under 1*/*2. However, vaccinating essential workers early allows far more of that fraction to be protected by vaccination, whereas in the oldest-first strategy more infection is required to achieve declining infections.

Using doses of vaccines effectively is particularly relevant where vaccine supply is low and rollout is slow—something likely to be relevant in low- and middle-income settings for many months to come. This urgency is amplified by the increasingly-recognized importance of long COVID and the severity and extent of chronic impacts that COVID-19 infection can have in adults of any age. This provides a counterpoint to what has become the conventional wisdom that age-based rollouts are the most effective at saving lives.

We modelled a rise in transmission 60 days into the simulation, in part because of increasing evidence that at the time of writing variants of concern (VOC) were rising in frequency [47,48] during vaccine rollout in Canada and similar jurisdictions and these VOC have increased transmissibility [49]. As their frequency rises, VOC are therefore likely to drive higher transmission rates, particularly in the current context where many areas are considering relaxing restrictions following declining cases and encouraged by the availability of vaccines. If VOC transmission is contained, the relaxation of measures itself is likely to result in higher transmission.

Even without considering Long COVID and COVID complications, vaccinating essential workers sooner has strong benefits in terms of reducing infections, hospitalizations, deaths, and in terms of net monetary benefit (NMB). However, taking chronic outcomes into account makes the advantages of prioritizing high-contact individuals even more stark, showing that they potentially save hundreds of millions of dollars of additional NMB (in a population of approximately 5M). These long-term consequences of COVID infection could impact the future health of 0.5% of the population under an oldest-first vaccination strategy, and far fewer (0.25%) if essential workers and/or younger adults are vaccinated earlier. Despite uncertainty in the likelihood and duration of longterm consequences of COVID infection, Long COVID and COVID complications need to be included in considerations of vaccine priority.

Our results about the relative benefits of different rollouts hold for jurisdictions where social distancing and other non-pharmaceutical interventions keep the basic reproductive number relatively low, and where vaccine supply is limited. There are several additional limitations. We have assumed that the vaccines in question are effective at reducing transmission as well as severe outcomes. Our results should not be applied to jurisdictions where these assumptions—about epidemic trajectory, vaccine supply and vaccine efficacy—do not hold. We have performed a sensitivity analysis to ensure that our results are robust to key unknown parameters (see S1 Text Supplemental Material). In some parameter regions, such as when *R* is very low (*R* = 1.15), the benefit conferred by vaccinating essential workers early is small, especially in terms of overall deaths. However, we note that there was no scenario or outcome considered in which such strategies performed worse than the oldest-first strategies. Furthermore, our calculations of the QALYs lost and NMB of different strategies were based on estimates of SMR, life expectancy at different ages, and other parameters specific to particular chronic conditions. All these parameters are subject to a great deal of uncertainty, and updating them as more information is available about mortality and morbidity due to COVID may change the total estimated cost of chronic conditions, but not the fact that such costs are large and need to be factored into consideration.

Strategies that explicitly target high-contact workers were adopted in several Canadian provinces [50,51]. These provinces additionally reserved first doses for younger adults ahead of second doses for older adults. Although it is difficult to assess the impact of vaccination strategies alone, since there are many additional factors affecting infection and mortality rates, our findings suggest that these vaccination strategies were key to the sustained and rapid decline in COVID-19 cases across Canada in late Spring–Summer, 2021. As of the time of writing, COVID-19 cases and deaths across the country remain low, even following significant re-opening and emergence of highly transmissible variants.

## Supporting information

S1 Text In the document supplemental material we provide more details about several aspects of our work, including model structure and parameters, our economic analysis, a validation of our model against data, and a sensitivity analysis. Within S1 Text we have:
Fig A. Model schematic.Table A. Demographic parameters in our model.Table A. Parameters we use for our economic analysis.Fig B. Savings for each strategy.Fig C. Results for alternative strategies.Fig D. Validation against historical data.Fig E. Results for varying the effect of vaccination against Long COVID.Fig F. Results for higher values of *R*.Fig G. Sensitivity with respect to *α* (the proportion of workplace contacts among nonessential workers).Fig H. Sensitivity with respect to *v*_*e*_ (the efficacy of vaccination against infection).Fig I. Sensitivity with respect to the proportion of essential workers.Fig J. Sensitivity of results with respect to randomly resampling the contact matrix.Fig K. Optimal strategy in terms of minimizing deaths, as a function of *v*_*e*_ and *v*_*p*_.Fig L. Optimal strategy in terms of minimizing deaths, as a function of *v*_*e*_ and the rate of vaccination.
(PDF)
