# Supplementary material for: Vaccine rollout strategies: The case for vaccinating essential workers early
Source: PLOS Glob Public Health. 2021 Oct 13;1(10):e0000020. doi: 10.1371/journal.pgph.0000020 (PMC10021761; doi:10.1371/journal.pgph.0000020)
Supplement: S1 Text — Within S1 Text we have: Fig A. Model schematic.Table A. Demographic parameters in our model.Table A. Parameters we use for our economic analysis.Fig B. Savings for each strategy.Fig C. Results for alternative strategies.Fig D. Validation against historical data.Fig E. Results for varying the effect of vaccination against Long COVID.Fig F. Results for higher values of R.Fig G. Sensitivity with respect to α (the proportion of workplace contacts among nonessential workers).Fig H. Sensitivity with respect to ve (the efficacy of vaccination against infection).Fig I. Sensitivity with respect to the proportion of essential workers.Fig J. Sensitivity of results with respect to randomly resampling the contact matrix.Fig K. Optimal strategy in terms of minimizing deaths, as a function of ve and vp.Fig L. Optimal strategy in terms of minimizing deaths, as a function of ve and the rate of vaccination. (PDF) [file pgph.0000020.s001.pdf]

S1 Supplemental Material for *Vaccine Rollout Strategies: The Case for Vaccinating Essential Workers Early*

N. Mulberry[1], P. Tupper[1], E. Kirwin[2,3], C. McCabe[2,4], and C. Colijn[1]

[1]Department of Mathematics, Simon Fraser University
[2]Institute of Health Economics, Alberta, Canada.
[3]Health Organisation, Policy, and Economics, School of Health Sciences, University of Manchester, United Kingdom.
[4]Department of Emergency Medicine, Faculty of Medicine and Dentistry, University of Alberta, Canada.

Model Structure

We model the transmission and progression of COVID-19 with a susceptible-exposed-infectious-recovered (SEIR) model, illustrated in Fig A. We include both vaccinated and unvaccinated individuals, and allow for some fraction of vaccinated individuals to become infected even after vaccination (according to the parameter v_e). However, among individuals who become infected after vaccination, they are nonetheless protected against adverse outcomes (according to parameter v_p). This model represents a variation on that of Bubar et al[1], with the major difference between this work and the original being in the essential worker contact structure and in the computation of the force of infection, λ. A discussion on the contact structure of the model is given below in the section titled **Contact Matrix**. The force of infection acting on a susceptible class in age group i is given by

$$\lambda_i = u_i \sum_j c_{ij} \frac{I_j + I_{vj} + I_{xj}}{N_j}$$

where u_i is the relative susceptibility of the ith group times an overall transmission parameter β (i.e. $u_i = \beta z_i$ with β adjusted to achieve the desired underlying effective transmission R). In addition, c_{ij} is the contact matrix, and I_j, I_{vj} and I_{xj} are the infectious individuals of group j who are unvaccinated, vaccinated post exposure to COVID-19, and vaccinated, respectively.

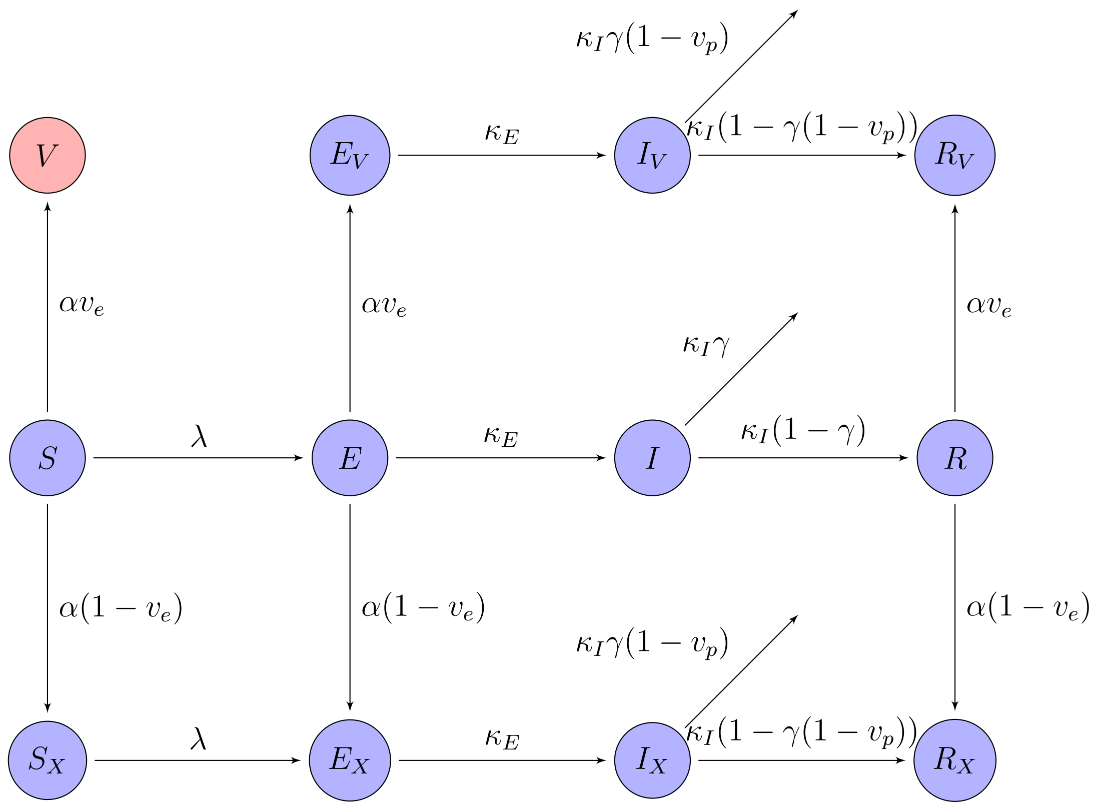

Figure A: Model schematic. The states are: susceptible (S), exposed (E), infected (I) and recovered (R). The total force of infection (which takes into account the contact structure) is denoted by λ, and κ_E, κ_I are the transition rates from the E and I states, respectively. On each day of the simulation, the entire non-infectious population is vaccinated at rate α. Following vaccination, a susceptible person is fully protected against infection (V) at a rate proportional to the vaccine efficacy, v_e. Otherwise, they remain susceptible to infection (S_X). The parameter γ represents the infection fatality rate among unvaccinated individuals, while v_p denotes the protection conferred to individuals who become infected following vaccination.

Model Parameters

For each vaccination scenario and choice of parameters, we measured multiple outcomes: number of infections, deaths, hospitalization, and cases of Long COVID. Whether any specific infection became a case of Long COVID was determined by an age-dependent probability which was computed using data from the Covid Symptoms Study App, (CSSA)[2]. The CSSA defines Long COVID as having symptoms longer than 28 days. Hospitalizations and deaths per detected case were estimated from data provided by the Public Health Agency of Canada[3], Table 13-26-0003, for infections detected in the period between Sept. 15, 2020 and Jan. 15, 2021. At this time, the ascertainment fraction (the fraction of COVID infections that are detected as cases) is believed to have been relatively high[4]. We use an ascertainment fraction of 0.75 to scale the estimated hospitalization and death rates per detected case to infections in the model, and compared the resulting hospitalizations and deaths (along with incidence) to data from British Columbia, Canada in fall

2020 (see Fig D).

A disproportionate number of deaths have been among those living in long term care (LTC) settings, accounting for 2/3 of British Columbia's deaths and 80% of Canada's death in the earlier part of the pandemic[5,6] despite the fact that well under 10% of Canada's seniors aged 65 and over are in long term care[7]. Long term care settings have been prioritized for vaccination and there are already indications of benefits. Accordingly, we reduced the infection fatality rate to reflect the fact that LTC settings are very unlikely to see death rates as high as they experienced in the pandemic to date; we reduced the rate by 1/3, consistent with the above numbers and the PHAC data on cases by age[8].

Age	Hosp rate	Death rate	Long COVID rate	Vaccine hesitancy	Prop. essential
0–9	0.0054	5.27E-05	0.04	NA	0.0
10–19	0.0054	5.27E-05	0.04	NA	0.0
20–29	0.0119	2.05E-04	0.04	0.3	0.17
30–39	0.0216	5.33E-04	0.08	0.2	0.20
40–49	0.0301	0.0014	0.15	0.2	0.17
50–59	0.0613	0.0028	0.25.	0.2	0.15
60–69	0.137	0.0163	0.25	0.15	0.16
70–79	0.2885	0.0775	0.25	0.15	0.10
80+	0.3279	0.213	0.25	0.15	0.0

Table A: Death, hospitalization, and Long COVID rates by age, per infection. Death and hospitalization rates are based on publicly available data from the Public Health Agency of Canada (PHAC; StatCan Table 13-26-0003)[3] and Public Health Ontario[9]. Death rates for ages 0–49 were taken from the Ontario data because the PHAC values were zero. The death rate among those $80+$ was adjusted to account for vaccination that has already taken place in long-term care. Rates of Long COVID were estimated from data in Sudre et al[2], where Long COVID is defined here to be symptoms for more than 28 days. The proportion of essential workers by age was taken from the COVID Speak survey[10].

Economic Analysis

For each death due to COVID at a given age QALYs lost are the number of expected years of life left for someone of that age but with two adjustments. Firstly, since those who die of COVID are more likely to have preexisting conditions that shorten one's life, we included a standardized mortality ratio[11] (SMR) of 2. Secondly, we discounted future years of life lost by 1.5% per annum, following the guidelines of CADTH[12]. We did not assume a reduction in baseline quality of life for individuals who died from COVID.

For the loss of QALYs due to acute infection we used figures from Kirwin et al[13]. Each infection was given an age-dependent loss of quality adjusted days (Table A). Hospitalizations incurred a utility decrement of 0.58, which was adjusted for mean duration of hospital stay by age group,

to obtain the quality adjusted days lost for a hospitalization. For those discharged from the hospital, an additional utility decrement of 0.1 QALY was applied for the one-year period following discharge, as individuals do no immediately return to their pre-COVID health state following discharge[13].

The QALY lost for chronic outcomes of COVID infection is the factor with the greatest uncertainty, since we have not been able to observe COVID survivors for multiple years. We started by estimating utility decrements for chronic outcomes of COVID (including those that fall under the rubric of Long COVID such as ME/CFS, and other complications). We identified all chronic conditions which have been observed at a higher rate among COVID hospitalization cases versus controls and collected utility decrements and annual incremental health system costs associated with each of these conditions. We weighted the collected values by the prevalence of each condition in the general population (pre-pandemic), and adjusted each estimate for duration of symptoms, survival, and discount rate. (For details see Supplemental Materials.) We estimated an annual utility decrement of 0.16. To approximate the (highly uncertain) future burden of chronic outcomes due to Long COVID and COVID complications, we assume that COVID infections that are severe enough to require hospitalization have a 20% chance of leading to a chronic outcome (whether it be ME/CFS or another) that has this utility decrement for whichever comes sooner, 25 years or the natural end of their life. This probably overestimates QALYs lost in some ways (maybe chronic outcomes improve after a few years, and/or perhaps they have more modest utility decrements) and an underestimate in others (one does not have to be hospitalized to get Long COVID). We then adjusted for currency and inflation, and discounted future years of life lost by 1.5% per annum as before. We intend this term to include all chronic outcomes of COVID-19 infections (both Long COVID and COVID complications) which likely include many conditions of varying frequency, duration, and severity.

We then determined a NMB (loss) for the pandemic, first by converting QALYs lost to a monetary equivalent value by multiplying by $30,000 Canadian dollar[12] and then adding estimates of costs due to hospitalization and chronic outcomes. Using estimates of the direct costs of hospitalisation from Kirwin et al[13] gave $1391.88 per day of hospitalization. The cost of chronic outcomes was again estimated by weighting the cost per year of the list of chronic conditions by their pre-pandemic prevalence, leading to a value of $10,019.88 per individual per individual annually.

Parameter	Value	Source
max duration of chronic outcome	25 years	
fraction of hospitalization leading to chronic outcome	0.2	
utility decrement of chronic outcome	0.16	see SM
cost of hospitalization	$1,391.88	[13]
cost of year of chronic outcome	$10,019.88	see SM
dollar value per QALY	$30,000	[12]

Table B: Parameters for economic analysis.

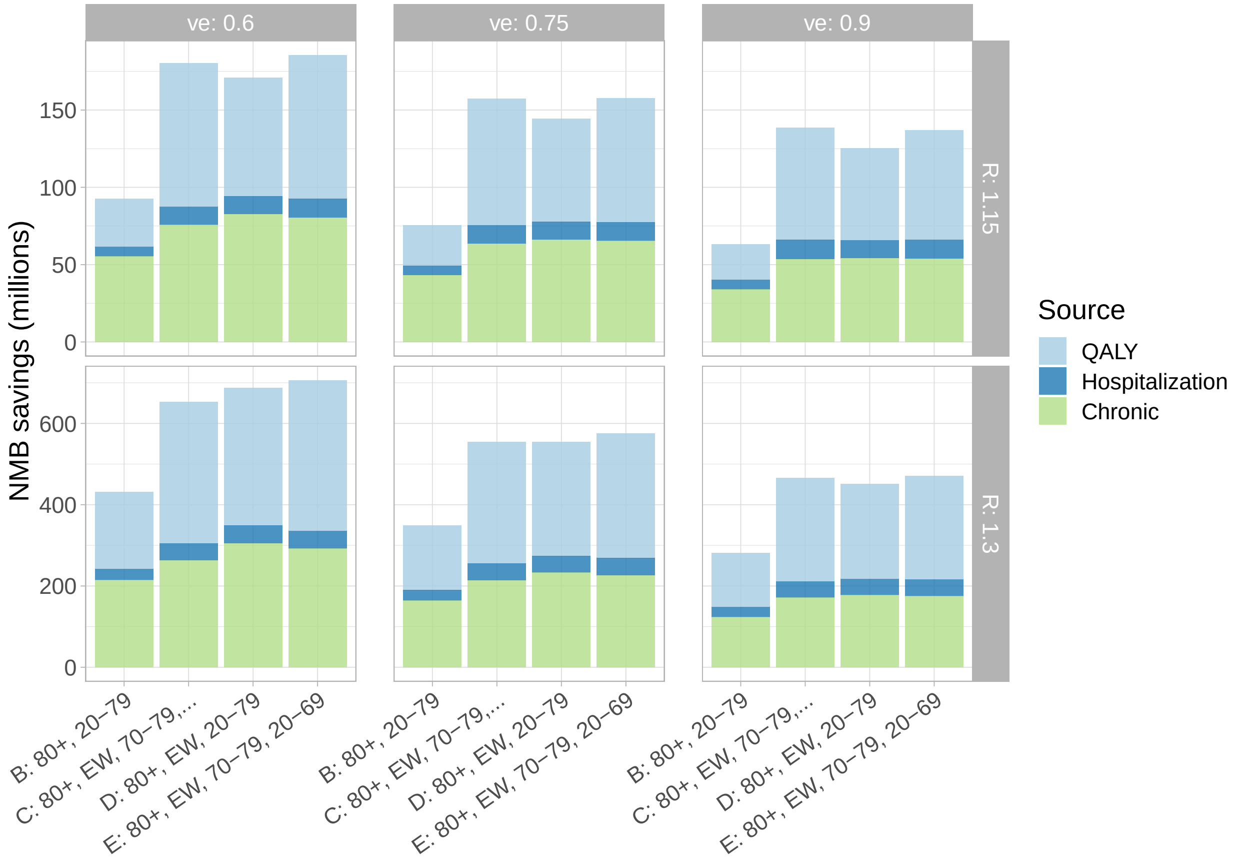

Figure B: Savings for each strategy in millions, compared to Oldest to Youngest (strategy A). The parameter v_e denotes the efficacy against transmission (top labels) and R is 1.15 (top) or 1.3 (bottom).

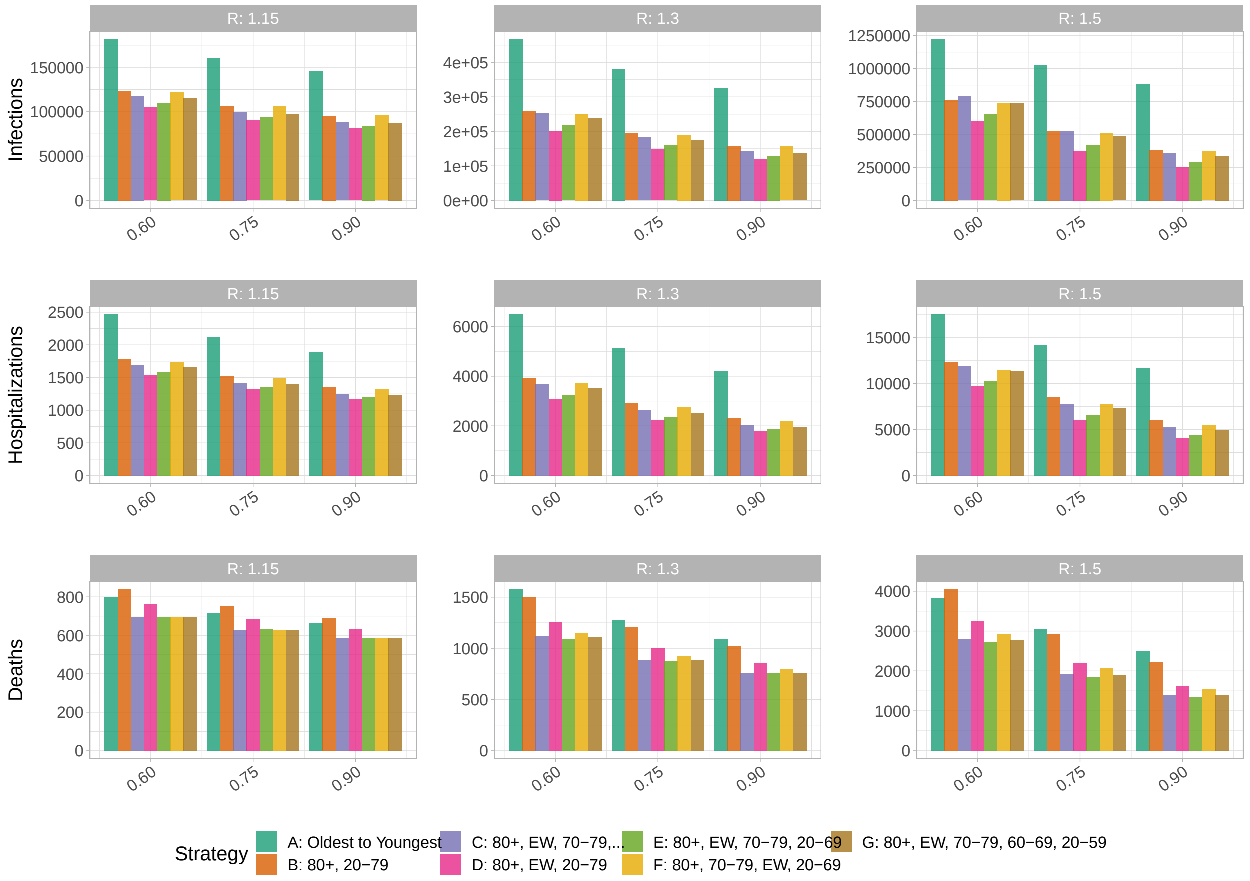

Figure C: Alternative strategies. Fixed $\alpha = 0$, $v_p = 0.9$.

Contact Matrix

To model contacts among all 15 groups, we first start with the four matrices developed by Prem et al[14] for estimated contact patterns among Canadians at home, work, school, and all other locations. We assume that the average contact rates among all Canadians reflect those among British Columbians. Each contact matrix provides an estimate of contact patterns over age groups 0–70+ in five-year increments. We first combine each age group into the desired ten-year bins using demographic data from British Columbia (BC), Canada. We then obtained survey data to estimate the distribution of essential workers by age group (the BC COVID-19 SPEAK Survey[10]); these proportions varied from 20% of 30–39 year-olds to approximately 10% of 70–79 years-olds. In total, we estimate that approximately 13% of the population of BC are considered to be "essential" workers. We use this data to split the working adult age groups into essential worker and non-essential worker compartments, assuming an even distribution of the number of contacts, following the technique of Buckner et al[15].

We model social distancing and other NPI measures by scaling these four contact matrices. First, we eliminate workplace contact between non-essential workers. Schools have remained open in BC, and so we do not eliminate school-based contact. The only asymmetry between essential

and non-essential workers is thus in the amount of workplace contact. We subsequently construct the total contact matrix by adding all of the school, work, home and other contact matrices. We subsequently compute the reproductive number of this “total” contact matrix using the Next Generation Method (as outlined in Bubar et al[1]). The contact matrix is scaled accordingly to achieve a prescribed value for R. Therefore, in our formulation, R reflects the extent and effectiveness of NPI measures (but does not reflect the impact of vaccination or immunity through natural infection). The underlying transmission (parameterized by R) impacts the relative benefits of different vaccination strategies, and we explore this impact.

Validation

In Fig D, we validate the model contact structure, and the age-based hospitalization and fatality rates against observed cases, hospitalizations, and deaths in British Columbia over a period from October 1 to December 1 2020.

Net Monetary Benefit relative to Oldest-to-Youngest

In Fig B we show the Net Monetary Benefit of each of the alternative vaccination strategies, relative to strategy A (vaccinating from oldest to youngest). The NMB of any of the strategies relative to no vaccinations at all is considerably larger[13].

Fig C explores two additional strategies that were not considered in the main text. Both of these additional strategies prioritize essential workers. Strategy F is similar to strategy E, except we consider allocating vaccines to 70–79-year-olds before essential workers (followed by a general rollout). Strategy G is again similar to strategies C and E, where we prioritize essential workers, then target older adults sequentially, followed by all 20–59-year-olds. We see that all of strategies C through G (which prioritize essential workers) out-perform the purely age-based strategies (A and B) in terms of infections, hospitalizations and deaths. However, the strategies which also prioritize adults aged 70–79 (strategies E–G) outperform strategy D (which does not prioritize this age group) in terms of mortality. Across outcomes, we observe that strategies E–G perform very similarly.

Efficacy of the vaccine against Long COVID

In our model, individuals with Long COVID do not continue to transmit COVID, and their numbers therefore do not affect the dynamics of the rest of the model. Here we illustrate the impact of assumptions about the vaccine’s efficacy against Long COVID.

The model in the main text used the same efficacy against Long COVID as for severe outcomes (0.9). Fig E illustrates that this efficacy has a minor outcome on the Long COVID prevalence. Although we explore an efficacy of 0.6 compared to the default of 0.9, so a 30% decrease in efficacy this has a minor impact (a less than 30% reduction in Long COVID), because most of the infections in these scenarios occur among those who were not vaccinated. Most of the Long COVID cases also, therefore, occur among the unvaccinated.

Sensitivity Analysis

We explore the sensitivity of our main result—that strategies which prioritize essential workers outperform age-based only strategies—to various model parameters. We show that our conclusion on prioritizing essential workers is robust (except in a small, and perhaps unrealistic parts of the parameter space), but that distinguishing among such strategies is often sensitive to various model parameters.

First, we explore transmission rates that so far seem unrealistic for the Canadian setting but have been used in other models[1]. Fig F shows trajectories where R is 1.5 or 2, vaccination proceeds at 0.4% per day with an efficacy against transmission of 0.6 and efficacy against disease is at 0.9 as in the main text.

Next, we explore the sensitivity of our results to α, the fraction of workplace contact occurring among nonessential workers (taken the be zero in the main text). Fig G shows that for values of R between 1.15 and 1.5, our conclusion that prioritizing essential workers outperforms an age-based rollout is robust to changes in α. However, as α increases the net-benefit of targeting essential workers (strategies C–E) over younger adults (strategy B) decreases (note that when $\alpha = 1$, all working age adults have the same level of contact, regardless of essential worker status). Therefore, the relative performance of strategies which target essential workers (strategies C–E) is sensitive to both R and α, although our conclusion that such strategies outperform general age-based rollouts is robust.

We similarly explore the sensitivity of our results to a broader range of v_e, the efficacy of vaccination against infection, in Fig H. We find that strategies which target younger adults sooner (strategies B and D) perform poorly in terms of deaths when vaccine efficacy is very low ($v_e < 0.5$) across the different R values. Strategies that target essential workers and also older adult age groups (strategies C and E) are robust even for low values of v_e.

We furthermore explore the sensitivity of our results to changes in the contact matrix structure. First, we consider changing the proportion of essential workers (Fig I). We find that strategies C and D, which prioritize essential workers and also older adults, are robust to changes in this frequency. However, strategy D (which does not prioritize older adults) is sensitive to changes in this frequency. Next, we find that our results are not qualitatively altered by random perturbations in the contact matrix (Fig J). However, these results highlight that we may not be able to use this model to determine the small differences among the benefits of strategies C–E (all of which target

essential workers, but differ in the details of the rest of the rollout).

Finally, in Figs K and L we test the relative performance of strategy C (which targets essential workers) against the age-based only strategies (A and B) over a wide range of parameters. These figures show the optimal strategy in terms of minimizing mortality for the given set of parameters. We find that strategy A (oldest to youngest) is only optimal in a very small region of the parameter space. Strategy C, which targets essential workers, is optimal almost everywhere. We note that we do not distinguish here across strategies D–G, which also target essential workers. These figures show that our conclusion that age-based only rollouts are non-optimal is robust except in parts of the parameter space which we consider to be unrealistic.

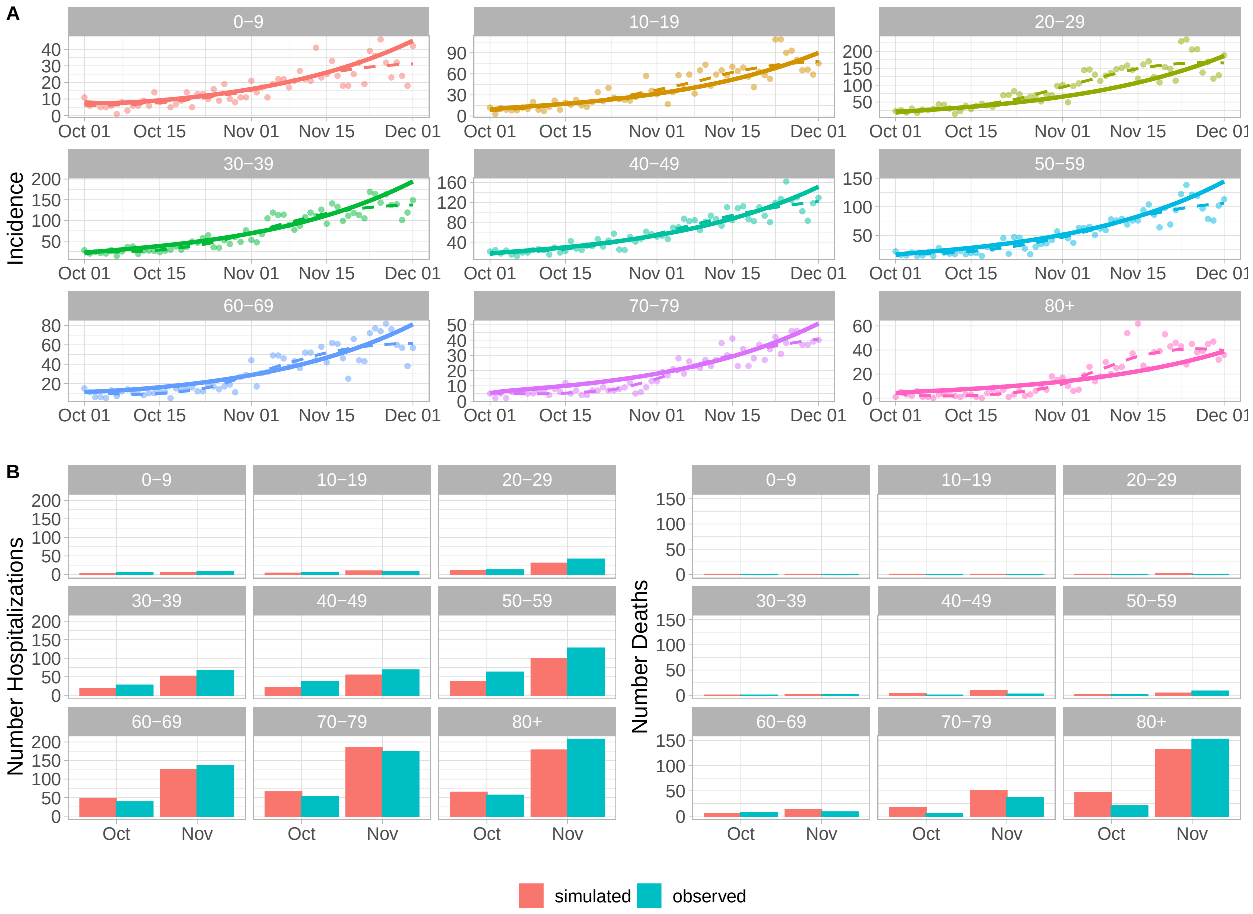

Figure D: Validation with the age distribution of cases, hospitalizations and deaths. A) Dots represent reported case counts by age; lines are simulated values. B) Bars represent total hospitalizations and deaths by month. Time period Oct-Dec 2020. Constant $R = 1.3$. Initial conditions taken from case count data.

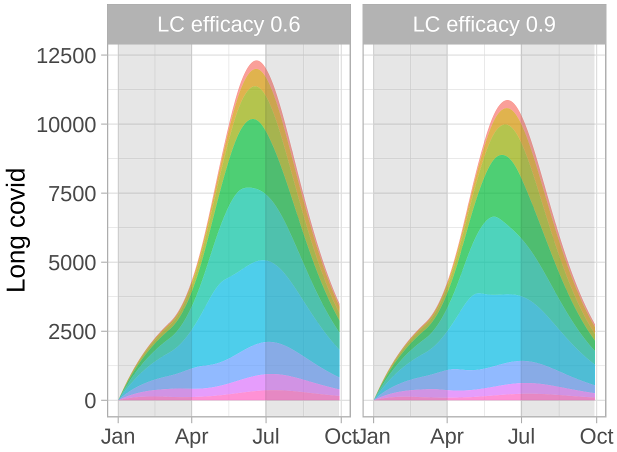

Figure E: The Long COVID prevalence over time is not impacted much by a substantial change in the vaccine's efficacy against Long COVID. In this demonstrative example, R is 1.05 during vaccination of those over 80, and then rises to 1.3 reflecting reopening or rising frequency of a higher-transmission variant. Colour indicates age as in Fig 1 of the main text.

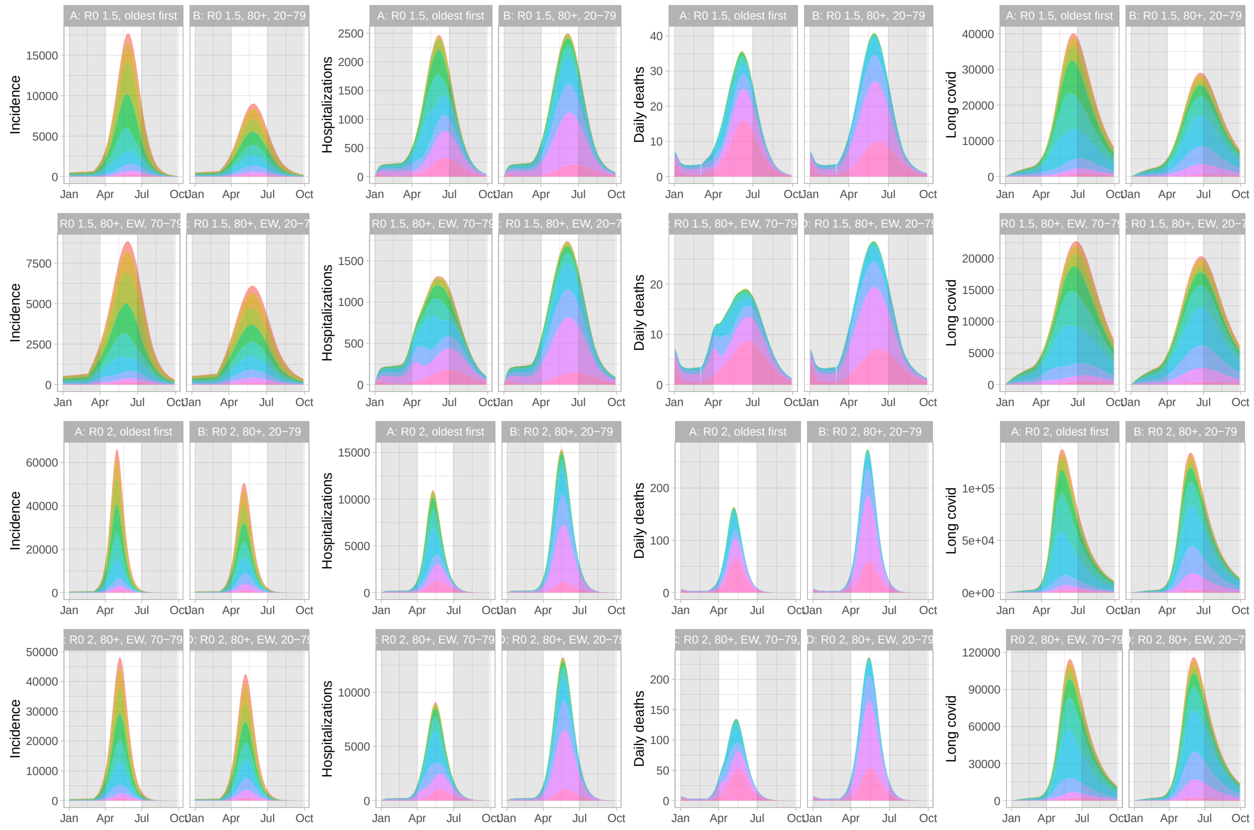

Figure F: Trajectories for a higher R value (1.5, 2). Here, vaccinating younger people sooner still has a benefit for infections and Long COVID, but results in higher deaths and hospitalizations. However, vaccinating essential workers in the context of an age-based rollout reduces all negative outcomes substantially when $R = 1.5$, and has limited impact when $R = 2$.

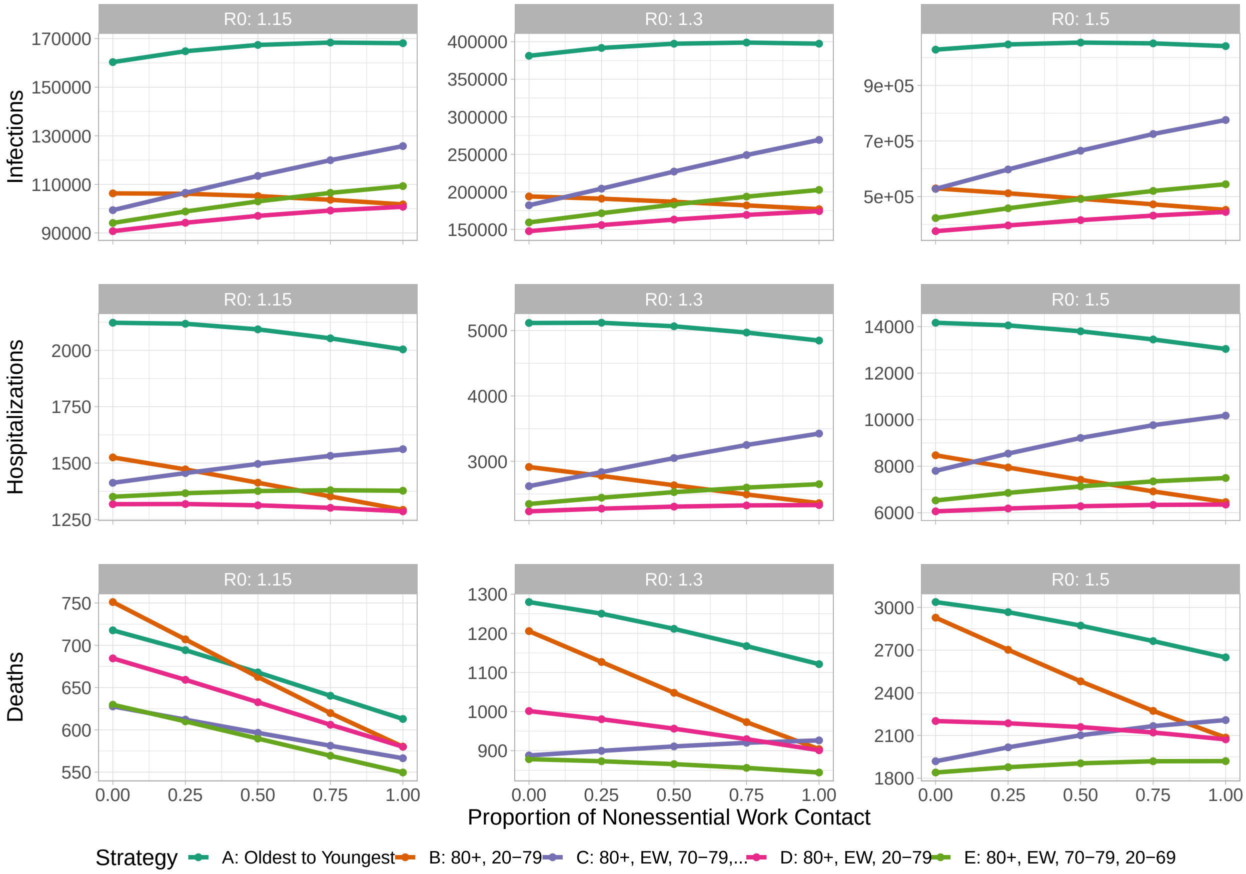

Figure G: Sensitivity with respect to α (the proportion of workplace contacts among nonessential workers). We vary R from 1.15 (left) to 1.5 (right). We fix $v_e = 0.75, v_p = 0.9$. The oldest-to-youngest strategy consistently performs worse than the others. The relative benefits of the other strategies are typically small and depend somewhat on α, though strategies including essential workers early (pink, green) consistently do well.

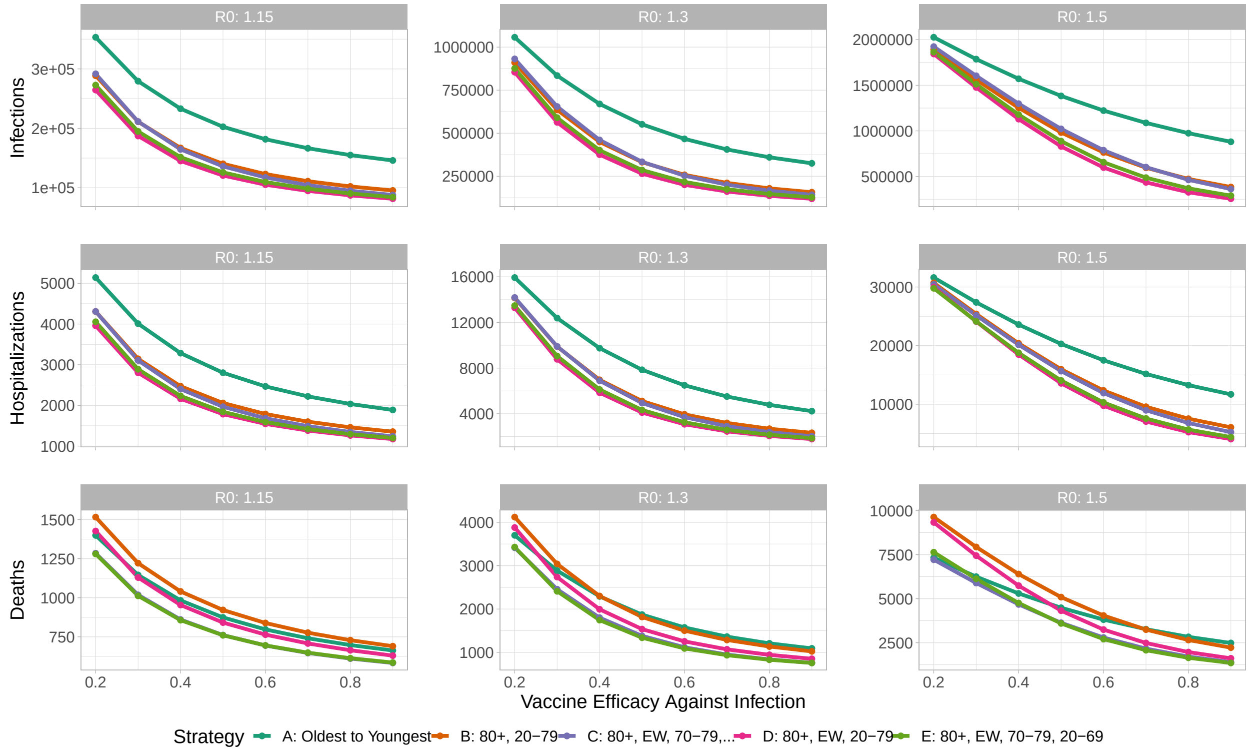

Figure H: Sensitivity with respect to v_e (the efficacy of vaccination against infection). We vary R from 1.15 (left) to 1.5 (right). We fix $\alpha = 0$, and $v_p = 0.9$.

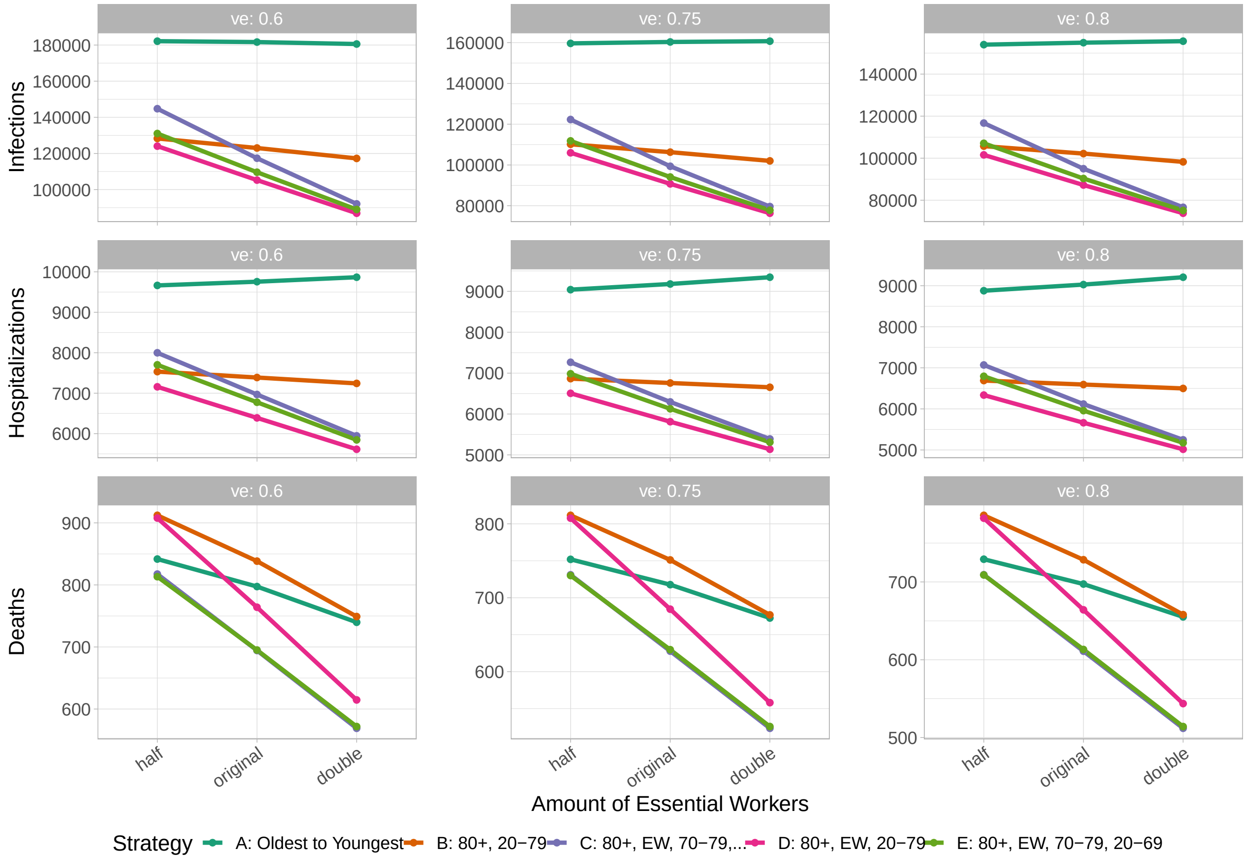

Figure I: Sensitivity with respect to the proportion of essential workers. We vary v_e from 0.6 (left) to 0.8 (right). We fix $\alpha = 0$, $v_p = 0.9$, $R = 1.15$. Similar results for other values of R. Note that strategies C and E often overlap.

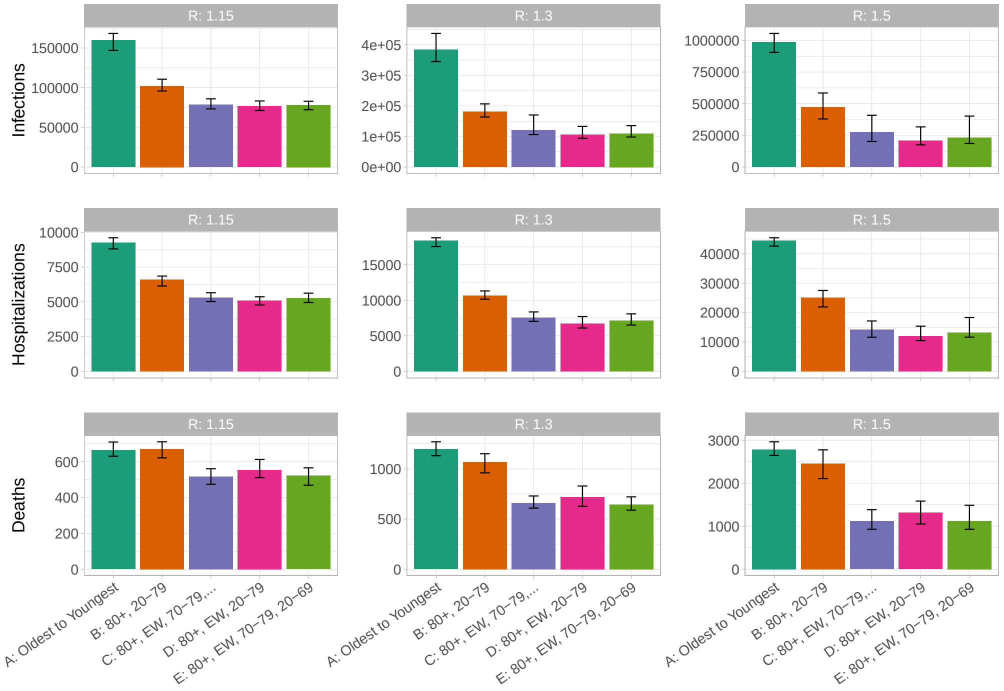

Figure J: Sensitivity of results with respect to randomly resampling the contact matrix. Bars indicate the median value, with 5 and 95% quantiles shown as error bars. Fixed $\alpha = 0$, $v_e = 0.6$, $v_p = 0.9$. For each simulation, perturb C by taking $\widetilde{C}_{ij} = C + \eta_{ij} C_{ij}$, where η is a symmetric random matrix with each entry drawn from a normal distribution with mean 0 and standard deviation 0.3. We take $\eta_{ij} = \eta_{ji}$ to preserve the contact structure of C.

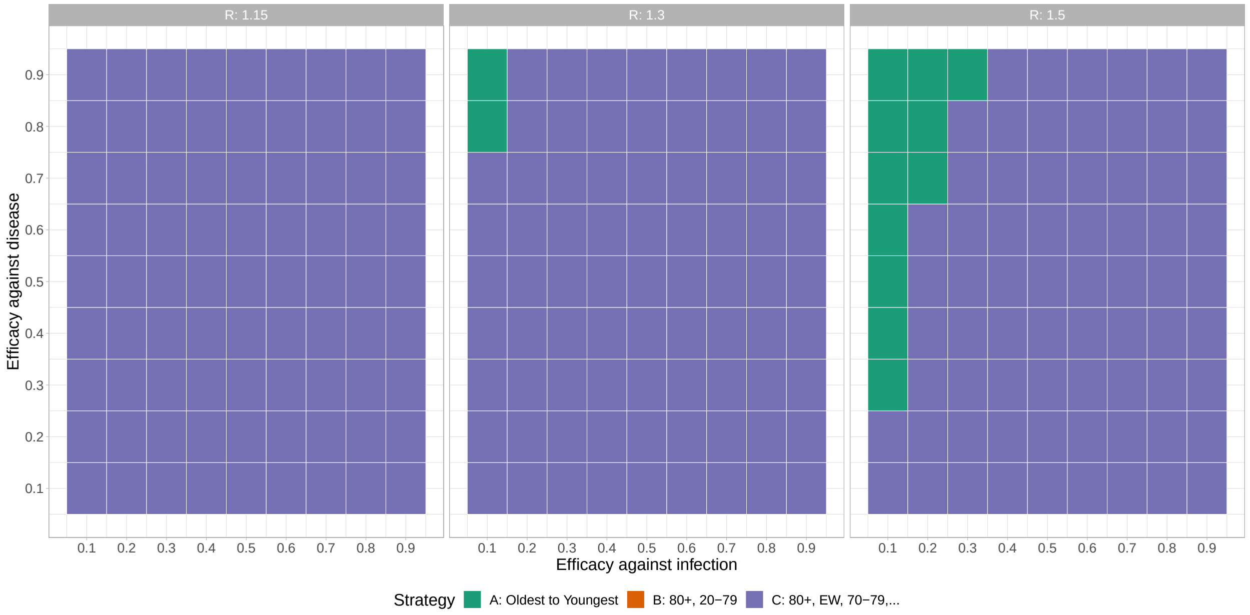

Figure K: Optimal strategy in terms of minimizing deaths, as a function of v_e and v_p. The rate of vaccination is fixed at 0.3 percent per day, and here we do not use a two-stage simulation. R is varied from 1.15 (left) to 1.3 (right). Note that other strategies which prioritize essential workers (Strategies D,E,F,G) often perform well also.

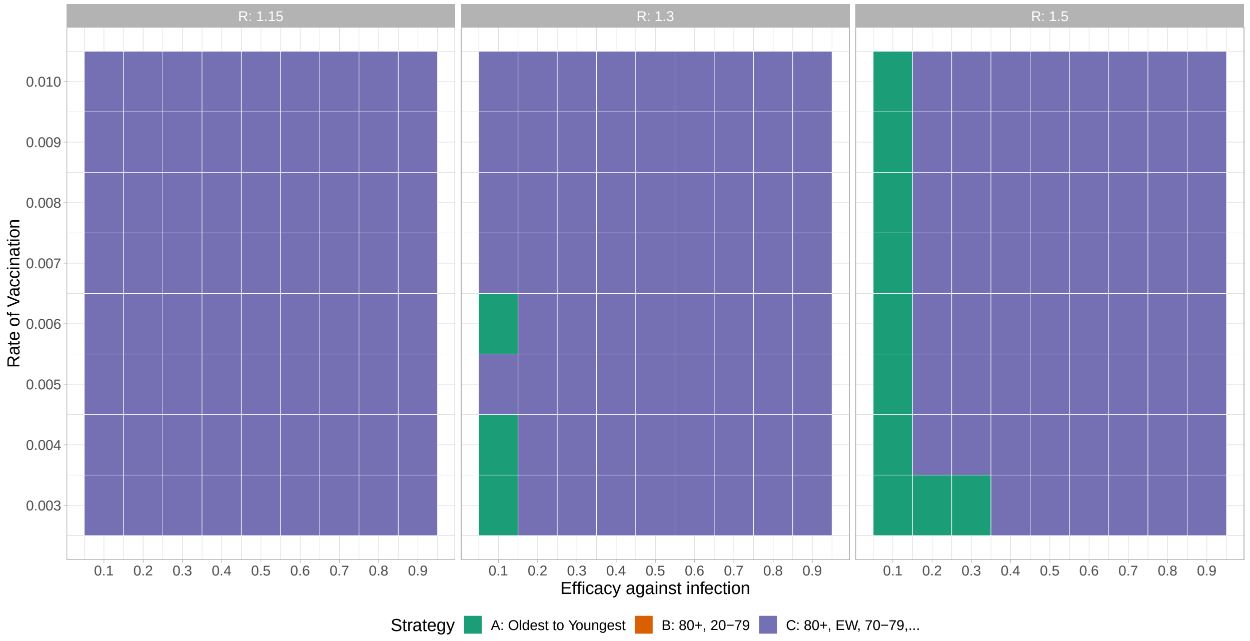

Figure L: Optimal strategy in terms of minimizing deaths, as a function of v_e and the rate of vaccination. Fixed $v_p = 0.9$, and here we do not use a two-stage simulation. R is varied from 1.15 (left) to 1.3 (right). Note that other strategies which prioritize essential workers (Strategies D,E,F,G) often perform well also. Vaccination rate is varied from 0.3% of the population per day to 1% of the population per day.

References

[1] Bubar KM, Reinholt K, Kissler SM, Lipsitch M, Cobey S, Grad YH, et al. Model-informed COVID-19 vaccine prioritization strategies by age and serostatus. Science. 2021.

[2] Sudre CH, Murray B, Varsavsky T, Graham MS, Penfold RS, Bowyer RC, et al. Attributes and predictors of Long-COVID: analysis of COVID cases and their symptoms collected by the Covid Symptoms Study App; 2020.

[3] Government of Canada, Statistics Canada. Preliminary dataset on confirmed cases of COVID-19, Public Health Agency of Canada; 2021. Accessed: 2021-2-17. https://www23.statcan.gc.ca/imdb/p2SV.pl?Function=getSurvey&SDDS=3233.

[4] Dougherty BP, Smith BA, Carson CA, Ogden NH. Exploring the percentage of COVID-19 cases reported in the community in Canada and associated case fatality ratios. Infect Dis Model. 2021;6:123–132.

[5] Migdal A. Residents in care facilities make up nearly two-thirds of COVID-19 deaths in B.C. CBC News. 2021 Jan.

[6] Webster P. COVID-19 highlights Canada's care home crisis. Lancet. 2021 Jan;397(10270):183.

[7] Long-term care and COVID-19: International comparisons;. Accessed: 2021-2-22. https://www.cihi.ca/en/long-term-care-and-covid-19-international-comparisons.

[8] Public Health Agency of Canada. From risk to resilience: An equity approach to COVID-19; 2020. Accessed: 2021-2-22. https://www.canada.ca/en/public-health/corporate/publications/chief-public-health-officer-reports-state-public-health-canada/from-risk-resilience-equity-approach-covid-19.html.

[9] COVID-19 Data and Surveillance;. Accessed: 2021-2-22. https://www.publichealthontario.ca/en/data-and-analysis/infectious-disease/covid-19-data-surveillance.

[10] COVID-19 Survey and Dashboard;. Accessed: 2021-2-22. http://www.bccdc.ca/health-info/diseases-conditions/covid-19/covid-19-survey.

[11] Briggs AH, Goldstein DA, Kirwin E, Meacock R, Pandya A, Vanness DJ, et al. Estimating (quality-adjusted) life-year losses associated with deaths: With application to COVID-19. Health Economics. 2020.

[12] Langley PC. True North: Building Imaginary Worlds with the Revised Canadian (CADTH) Guidelines for Health Technology Assessment. INNOVATIONS in pharmacy. 2017;8(2).

[13] Kirwin E, Rafferty E, Harback K, Round J, McCabe C. A Net Benefit Approach for the Optimal Allocation of a COVID-19 Vaccine. medRxiv. 2020. Available from: https://www.medrxiv.org/content/early/2020/12/02/2020.11.30.20240986.

[14] Prem K, Cook AR, Jit M. Projecting social contact matrices in 152 countries using contact surveys and demographic data. PLoS computational biology. 2017;13(9):e1005697.

[15] Buckner JH, Chowell G, Springborn MR. Dynamic prioritization of COVID-19 vaccines when social distancing is limited for essential workers. Proc Natl Acad Sci U S A. 2021 Apr;118(16).
